# Geochemical alkalinity and acidity as preferential site-specific for three lineages liverwort of *Aneura pinguis* cryptic species A

**DOI:** 10.1038/s41598-021-83553-x

**Published:** 2021-02-22

**Authors:** Alina Bączkiewicz, Jean Diatta, Maria Drapikowska, Patrycja Rodkiewicz, Jakub Sawicki, Monika Szczecińska, Katarzyna Buczkowska

**Affiliations:** 1 Department of Genetics, Adam Mickiewicz University, ul. Uniwersytetu Poznańskiego 6, 61-614 Poznań, Poland; 2 Department of Agricultural Chemistry and Environmental Biogeochemistry, Poznan University of Life Sciences, ul. Wojska Polskiego 71F, 60-625 Poznań, Poland; 3 Department of Ecology and Environmental Protection, Poznan University of Life Sciences, ul. Piątkowska 94C, 60-649 Poznań, Poland; 4 Department of Botany and Nature Protection, University of Warmia and Mazury in Olsztyn, ul. Łódzki Plac 1, 10-728 Olsztyn, Poland

**Keywords:** Ecology, Genetics, Biogeochemistry, Environmental sciences

## Abstract

The study focused on the verification of the preferential site-specific concept hypothesizing, that mineral elements could be playing an initiating role in the biological speciation within *Aneura pinguis* cryptic species. *A. pinguis* species A and soil materials were collected from three ecological sites of Poland. They underwent genetic (*Aneura pinguis*) and chemical analyses (soil materials) for pH, total and water soluble (active) forms of Ca, Mg, K, Na fractions. Data revealed trends in the site preference of three genetic lineages (A1, A2 and A3) of *A. pinguis* cryptic species A. Lineage adaptability index Ca/(Mg + K + Na) reflecting the dynamic character of site pH implied, that lineages A1 and A2 were both calciphilous. The A3 lineages were intrinsically acidophilous and this characteristics was also observed at some A1 lineages. Site concentrations of Ca and in some cases Mg too were crucial in shaping pH, but this process could have been controlled by each mineral element, individually. Calciphilous or acidophilous *A. pinguis* species may be “remotely” attracted by high or low Ca (or Mg) concentrations, for alkalinity or acidity emergence, respectively. Mineral richness at investigated ecological sites has possibly initiated opportunistic and specific site colonisation by *A. pinguis* lineages.

## Introduction

*Aneura pinguis* (L.) Dumort is a thalloid liverwort. It is common on all continents except Antarctica^1,2^. At the turn of XX/XXI century, genetic studies revealed that *A. pinguis* is a complex of cryptic species^3,4^ characterized by the lack of morphological alterations connected with significant genetic differentiation and reproductive isolation^5^. Biological studies indicated, that genetic distances between cryptic species of liverworts are at least as large as for taxonomically recognized species^6,7^. Before the discovery of cryptic species in *A. pinguis*, it was thought that slight morphological differences within these species were attributed to their plasticity^8^. The reason was due to simplified morphology and lack of distinct features that allow identifying species. Cryptic species of *A. pinguis* can be identified by genetic studies such as: isozyme markers^3^, ISSR markers^9^ or by barcode DNA sequencing^4,10^. So far, 10 cryptic species marked from A to J have been revealed within *A. pinguis.* The latter ones differ in geographic distribution and ecological preferences^3,4^.

Some cryptic species of *A. pinguis* additionally show marked genetic intraspecies differentiation dividing the species into subgroups (lineages). Genetic differences between subgroups are too small to recognize them as separate species. The highest intraspecies variation was detected in *A. pinguis* cryptic species A, where 3 separated lineages (subgroups) labeled A1, A2 and A3 were found. In Poland, all lineages occur in the Carpathians however may coexist in the same geographic region but it is rather rare. Lineages A1 are characteristic to the Pieniny Mts., A2 for Beskidy Mts., and for A3 the Tatry Mts predominate. The observed pattern of genetic diversity, geographic distribution and ecological preferences of A*. pinguis* cryptic species A implies that the speciation in these species results possibly from the specialization in adaptation to different habitat, where the soil plays a leading role.

Adaptability of bryophytes to specific habitats was broadly investigated and still, no full compromise has been retained^11–15^. Growth conditions and ecological as well as preferential features of bryophytes require additional and extensive research involving jointly genetic parametrisation and soil (growth substrata) characteristics, particularly for genetic lineages^16^. The calcareous, rocky and also organic characteristics of the soil or soil-like materials are up to date not closing the debate about the dynamic biodiversity processes of species like *A. pinguis*.

Scarce or even no scientific reports evaluated the potential relationship emerging between some mineral elements in soils and the potential colonisation by given bryophytes at *in-situ* level. On the other hand, studies under controlled conditions may supply with data indicative of probable biological evidence of site preference induced by calcium, for instance^11^. Next, soil chemical characteristics responsible for shaping pH should not be restricted to Ca solely. Ground interactions along with magnesium (Mg), potassium (K) as well as sodium (Na) are expected to reflect the dynamic character of site pH. Then, the highest the aqueous concentrations of alkaline elements (raise in alkalinity), the lowest the levels of acidic elements (H, mainly) expressing a decrease in acidity. Are alkaline elements and the resulting pH enough for elucidating the calciphilous and acidophilous characters of *A. pinguis*? Field research without genetic characteristics^15,16^ lead most frequently to pointing out at some interactions and trends, but doubtful statements emerge.

The current study has been specifically scheduled in order to outline some chemical characteristics of the soil materials supporting growth requirements of three lineages A1, A2, A3 of *A. pinguis* cryptic species A. Authors have elaborated an outstanding sampling method for soil materials and liverworts, which enabled the verification of the preferential site-specific concept. Data of this study focus on the hypothesis, that mineral elements expressed as alkalinity and acidity play an initiating role in the speciation within *A. pinguis* cryptic species A.

## Materials and methods

### Description of sampling sites

The sampling site covered three regions i.e., Pieniny Mts. (PNN), Beskidy Mts. (BS) and Tatry Mts. (T), located at the southern part of Poland. More details are listed in Table 1.

**Table 1:** Regions, localities and sample numbers of three genetic lineages (A1, A2, A3) within cryptic species of *A. pinguis* species A.

Region	Localities	Sample numbers			Geographic coordinates
		A1	A2	A3	
Pieniny Mts. (PNN)	Barbarzyna meadow	PPN 2–9			49° 25^′^ N; 20° 20′ E
	Górzańska wet meadow	PPN 7–5			49° 42′ N; 20° 39′ E
	Źródlisko Olesówka, source of the stream Łonny	PPN 11–5			49° 43′ N; 20° 43′ E
	Stream Łonny	PPN 13–2			49° 44′ N; 20° 41′ E
	Kąty settlement	PPN 16–2			49° 41′ N; 20° 37′ E
Beskidy Mts. (BS)	Wądołowy stream	BS12-14			49° 27′ N; 20° 25′E
	Kozłecki stream	BS 1–11	BS 7–9		49° 27′N; 20° 27′E
	Szczawa stream		BS3-28		49° 27′N; 20° 26′ E
	Wygon stream		BS 4–9		49° 26′ N; 20° 26′ E
Tatry Mts. (T)	Olczyska Valley	T 212–2			49° 16′N; 19° 59′E
	Wielka Sucha Dolina Valley			T 154–8 T 154–9	49° 16′ N; 19° 49′ E
	Dolina Suchej Wody Valley			T 180–13	49° 16′ N; 20° 01′ E
	Dolina Białego Potoku Valley			T 189–4	49° 16′ N; 19° 57′ E
	NE Skupniów Upłaz slope			T197-2 T 200–5	49° 15′ N; 20° 00′ E
Total		8	3	6	

**Figure 1:**
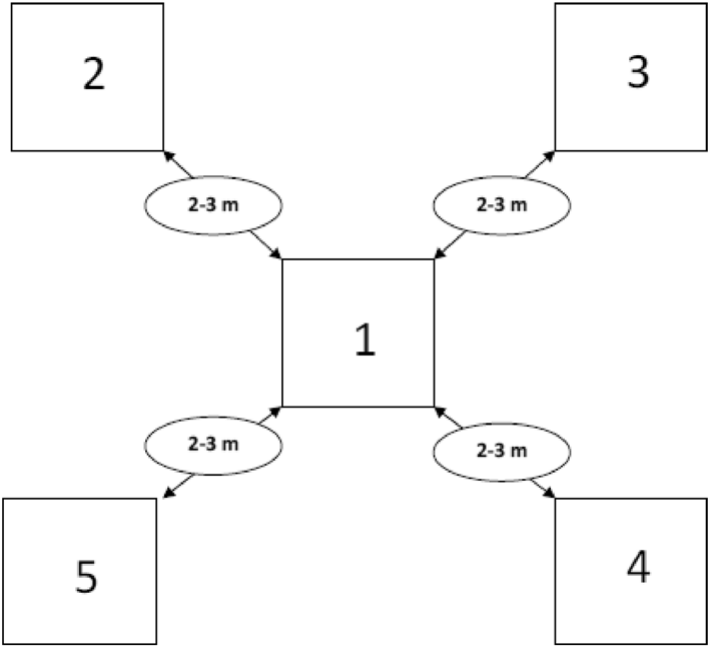
Sampling schedule elaborated for cryptic species of *A. pinguis* and soil materials (Authors).

Samples of soil materials (90) under *A. pinguis* cryptic species A. have been collected (layer was 0–5 cm) from 17 sites according to the scheme (Fig. 1). The materials have been dried at ambient room temperature (23–24 °C) for about 10 days, ground and passed through a 0.50 mm mesh sieve. Next, the whole soil materials were stored in PE bags, tightly sealed before chemical analyses.

### Biological analysis of *A. pinguis* species A

**Figure 2:**
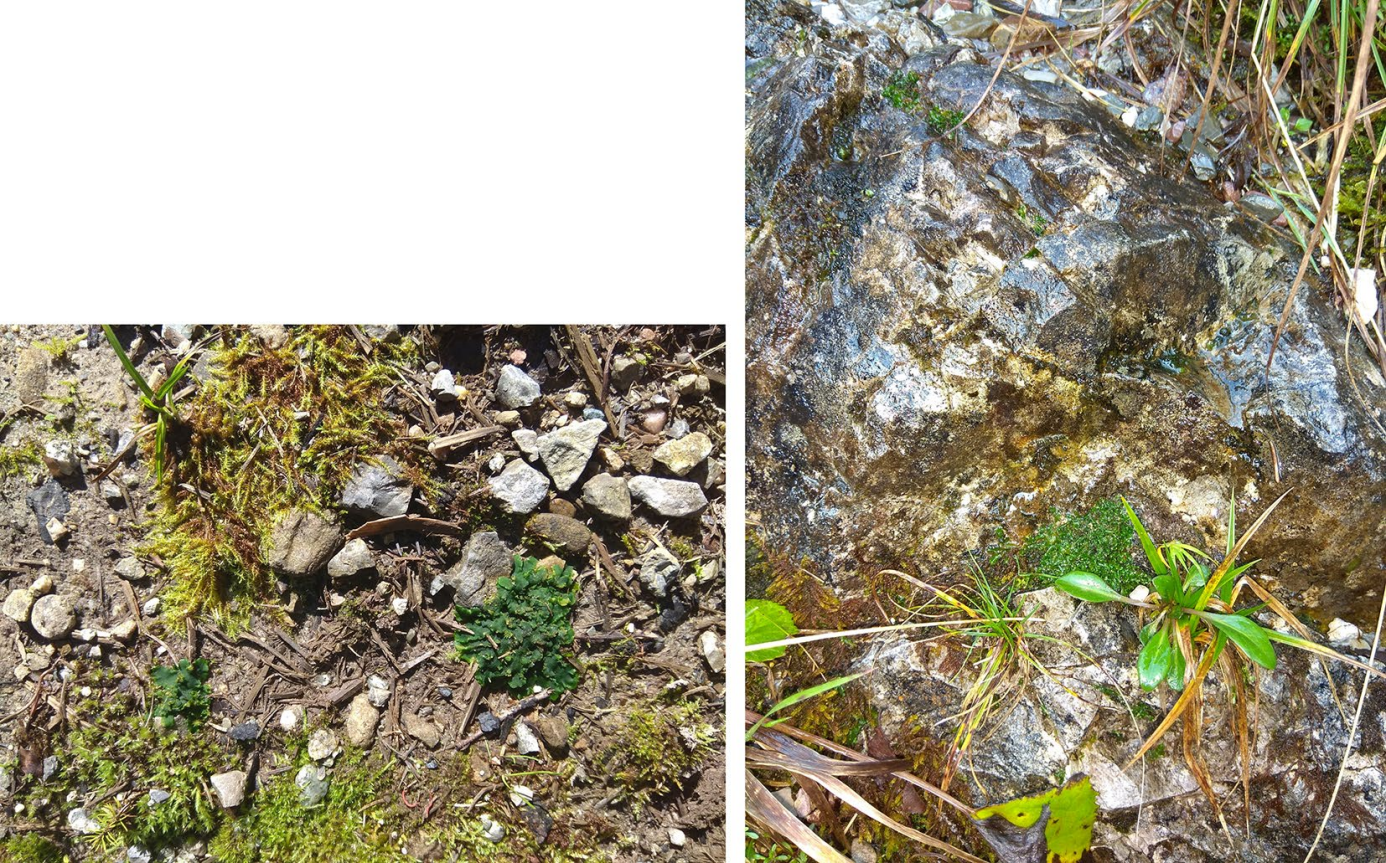
Mixed preferential ecosystems of *A. pinguis* cryptic species A. More soil material over rocky (left) as compared to more rocky over soil material (right).

Cryptic species of *A. pinguis* and genetic lineages (A1, A2, A3) within *A. pinguis* cryptic species A were identified on the basis of DNA sequences from five chloroplasts (*rbcL-a, matK, rpoC1, trnL-F, trnH-pabA*) and one (ITS1-5.8S-ITS2) nuclear genomes. The procedure of extraction DNA, primes, amplification and sequencing of examined regions was described in Bączkiewicz et al.^4^. The obtained DNA sequences were compared with the sequences of *A. pinguis* from the GeneBank^4^ and marked on this basis (Fig. 2).

### Chemical analysis of soil materials

- pH and active fractions of alkaline elements
  Soil materials were tested for pH, potentiometrically in aqueous slurry at the ratio 1:5^17^. The mixture was shaken on a rotative shaker (120 rpm) for 1 h, left to equilibrate for one hour before performing the measurements. After pH readings, the supernatant was thouroughly filtered for the determination of Ca, K, Mg and Na. The relevant concentrations were expressed as *active* fractions.
- Determination of total content of alkaline elements
  The total contents of Ca, K, Mg, Na in soil materials were assayed according to Gupta et al.^18^. Air-dried samples (1.00 ± 0.001 g) were weighed into a glass Erlenmeyer flask and 15 cm^3^ of 6 mol HCl dm^−3^ were added. Next, the mixture was heated on a sand-bath at 140 °C for 2 h under reflux. After cooling, it was filtered through a filter paper into 15 cm^3^ test tubes and filled up to the mark with bidistilled water.
  All chemical tests were replicated twice. The concentrations of Ca, K, Mg, Na were determined by atomic absorption spectrometry (AAS; Varian SpectrAA 250 plus, Varian Inc., Palo Alto, 143 Calif., USA). The relative standard deviation (RSD) was calculated from pooled data for applied methods. In the precision test, the average RSD (%) for all trace metals in particular tests (i.e. total, reactive) ranged from 0.76 to 1.50%. The accuracy was determined using a reference material [Estuarine sediment 277 CRM certified by the Bureau Community of Reference (BCR), Brussels, Belgium].

### Statistical analysis

- Biological materials
  The chromatograms of DNA sequences were edited and assembled using Geneious R6 (Biomatters, USA). Contigs were aligned using ClustalW as implemented in MEGA 6.06^19^. To illustrate differences between the analysed samples, neighbor joining trees were computed for individual and combined DNA regions using a TBR algorithm in MEGA 6.06. Neighbor joining trees were generated based on the Kimura 2-parameter model (K2P)^20^. Next, phylogenetic trees were generated by maximum likelihood (ML) method using MEGA 6.06 program. As supplementary measure of distinctiveness, the percentage of fixed nucleotide differences among samples was computed. A statistical significance of clades within inferred trees was evaluated using the bootstrap method with 1000 replicates. *Aneura maxima* was used as an outgroup in DNA analysis, but DNA sequences were obtained from the GenBank^4^.
- Soil materials
  Data were statistically evaluated by using STATISTICA 13.1 for Windows software^21^. The significance of the difference between mean values of active forms of alkaline elements (Ca, Mg, K, Na) and pH between growth sites of lineages A1, A2 and A3 were tested by analysis of variance (ANOVA) with Scheffe test by STATISTICA 13.1. ANOVA was used for normal distributed variables with repeatable measurements. Principal component analysis (PCA) was applied for investigating relationships between individuals from different populations, without any a priori assumptions^22^.

## Results

### Differentiation within *A. pinguis* cryptic species A

**Figure 3:**
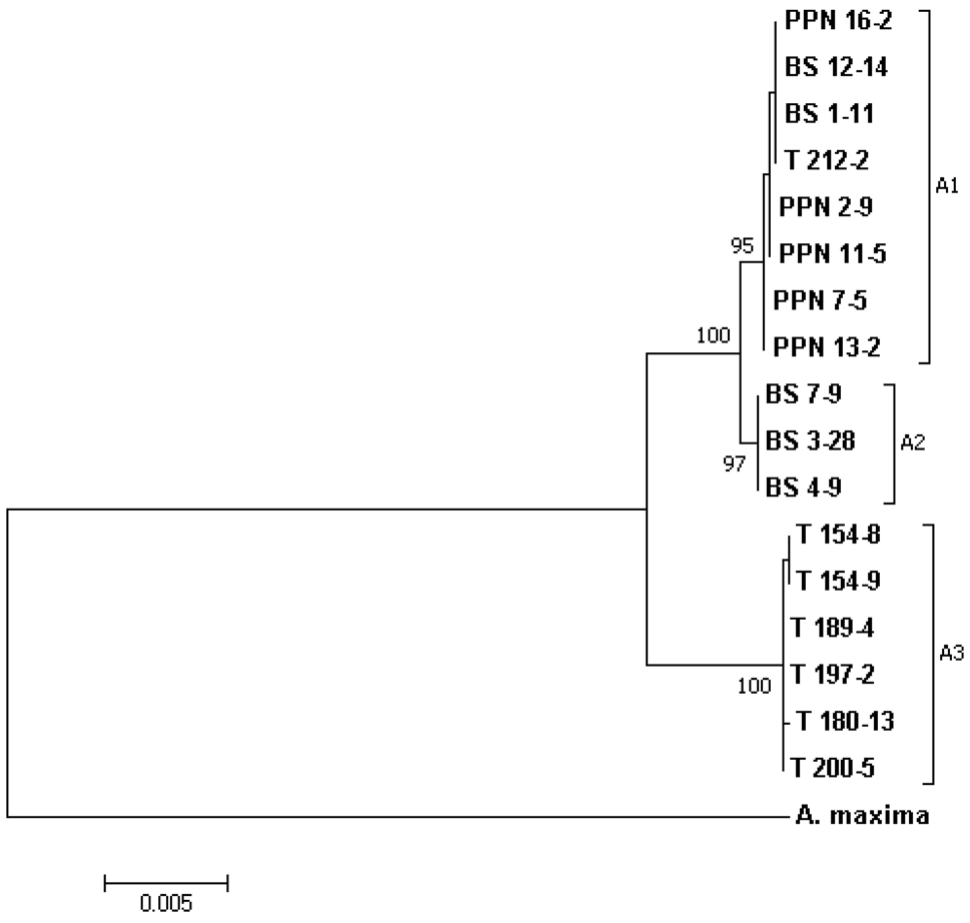
Phylogram resulting from maximum likelihood (ML) analysis based on combined data of all sequences and showing genetic similarity and differentiation between lineages A1, A2 and A3 of *A. pinguis* cryptic species A. Bootstrap values are given at branches. *A. maxima* was used as an outgroup for tree rooting.

Genetic studies using combined DNA sequences from five chloroplasts (*rbcL-a, matK, rpoC1, trnL-F, trnH-pabA*) and 1 (ITS) nuclear genomes (4598 bp) showed some differentiation within the *A. pinguis* cryptic species A into three distinct groups (lineages) A1, A2 and A3 (Fig. 3). Most of investigated plants belonging to the lineage A1 originated from the Pieniny Mts. (PNN), however two samples A1 were collected at the Beskidy Mts. (BS) and one at the Tatry Mts. (T). All plants identified to A2 and A3 came from the Beskidy Mts. and the Tatry Mts., respectively. Maximum parsimony analyses of combined plastid loci and the nuclear ITS locus produced trees showing that the lineage A3 is genetically the most distinct, while A1 and A2 reveal more similarity. In the K2P mode, the percentage of variation in the sequences between lineages A1 and A2 equals to 0.20%, while for A1 and A3 it raised five times, i.e. 1.0%. The same occurred for the lineages A2 and A3, 1.0%.

### Total and water soluble (active) alkaline elements

The total content of alkaline elements (Table 2) shows that the content of calcium (Ca) prevails over magnesium (Mg), potassium (K) and sodium (Na) at any investigated site, i.e. Pieniny Mts. (PNN), Beskidy Mts. (BS) and Tatry Mts. (T). Soil samples collected under the lineage A1 covered the whole three geographical distributions, where the site BS exhibited the highest Ca concentrations (31 459.6 mg kg^−1^) followed by PNN (16 178.8 mg kg^−1^) and finally T with 7 398.7 mg kg^−1^. Interestingly, the lineage A2 occurred only at the site BS characterised by high Ca content (27 303.4 mg kg^−1^), whereas A3 at the Tatry Mts. (T) where the level 22 227.6 mg kg^−1^ was recorded. These data imply that the lineage A1 may have developed site-specific adaptation mechanisms to various concentrations of calcium. In the case of A2 and A3, the observed Ca concentrations amounted to 27,303.4 and 22,227.6 mg kg^−1^, respectively and should be described as high.

**Table 2:** Total content of alkaline elements (Ca, Mg, K, Na) in the growth media of *A. pinguis* cryptic species A genetic lineage A1, A2, A3 at Pieniny, Beskidy and Tatry Mts.

Site	Descriptive statistics	Ca			Mg			K			Na		
		A1	A2	A3	A1	A2	A3	A1	A2	A3	A1	A2	A3
		mg kg^−1^			mg kg^−1^			mg kg^−1^			mg kg^−1^		
Pieniny Mts. (PNN)	***Mean***	***16 178.8*** (n = 25)*			***3 767.0*** ***(n = 25)***			***152.6*** ***(n = 25)***			***109.7*** ***(n = 25)***		
	SD	18 180.7			1 151.4			78.7			48.6		
	CV(%)	112.4			30.6			37.8			52.2		
Beskidy Mts. (BS)	***Mean***	***31 459.6*** ***(n = 10)***	***27 303.4*** ***(n = 15)***		***5 443.3*** ***(n = 10)***	***4 929.3*** ***(n = 15)***		***112.9*** ***(n = 10)***	***145.8*** ***(n = 15)***		***105.8*** ***(n = 10)***	***55.3*** ***(n = 15)***	
	SD	9 078.8	14 652.7		1 324.3	743.1		20.9	42.8		5.35	50.7	
	CV(%)	28.9	53.7		24.3	15.1		22.3	29.3		14.6	71.5	
Tatry Mts. (T)	***Mean***	***7 398.7*** ***(n = 5)***		***22 227.6*** ***(n = 30)***	***5 863.0*** ***(n = 5)***		***8 643.8*** ***(n = 30)***	***139.7*** ***(n = 5)***		***160.6*** ***(n = 30)***	***99.8*** ***(n = 5)***		***86.1*** ***(n = 30)***
	SD	7 517.7		13 812.7	2 286.2		2 129.5	32.6		40.3	11.8		44.6
	CV(%)	101.6		62.1	39.0		24.6	23.3		25.1	11.8		51.9

*Number of investigated samples.

Variations in magnesium (Mg) concentrations for the lineage A1 followed another pattern differing from that observed in the case of calcium. Its contents varied accordingly: T > BS > PNN, with the highest levels recorded for A3 and A1 at the Tatry MTs. (T), respectively. It should be mentioned that both Ca and Mg are in most cases responsible (Ca much more) for geochemical reactions controlling the pH of the growth media. The role of potassium (K) as well as sodium (Na) is generally less pronounced in these reactions, but also their contents, which were very low appeared as the proof.

The evaluation of site-specific occurrence of the lineages A1, A2 and A3 should not be performed on the basis of total content solely of alkaline elements, since this fraction is mostly informative on the current status of Ca, Mg, K and Na. Therefore, we have tested the soil samples for recovering the concentrations expressed as active fractions (Table 3) potentially involved in the growth process of these lineages. The levels (percentage share into the total content) of active Ca are significantly low and varied as follows: PNN (3.27%) > T (0.89%) > BS (0.73%) for A1, but raised to 1.34% (BS) in the case of A2. The lineage A3 has recorded a concentration of 0.71%, slightly comparable to A1, but at the same site (T).

**Table 3:** Content of active forms of alkaline elements (Ca, Mg, K, Na) in the growth media of *A. pinguis* cryptic species A genetic lineage A1, A2, A3 at Pieniny, Beskidy and Tatry Mts.

Site	Descriptive statistics	Ca			Mg			K			Na		
		A1	A2	A3	A1	A2	A3	A1	A2	A3	A1	A2	A3
		mg kg^−1^			mg kg^−1^			mg kg^−1^			mg kg^−1^		
Pieniny Mts	***Mean***	***529.2*** (n = 25)*			***95.4*** ***(n = 25)***			***37.9*** ***(n = 25)***			***22.7*** ***(n = 25)***		
	SD	481.8			84.5			47.2			13.8		
	CV(%)	91.1			88.6			124.6			60.9		
Beskidy Mts	***Mean***	***229.4*** ***(n = 10)***	***366.9*** ***(n = 15)***		***54.3*** ***(n = 10)***	***38.9*** ***(n = 15)***		***16.3*** ***(n = 10)***	***12.4*** ***(n = 15)***		***15.2*** ***(n = 10)***	***24.0*** ***(n = 15)***	
	SD	74.8	343.4		22.0	27.2		7.7	4.32		2.7	11.7	
	CV(%)	32.6	93.6		40.5	69.8		46.9	34.9		17.5	48.8	
Tatry Mts	***Mean***	***66.0*** ***(n = 5)***		***157.6*** ***(n = 30)***	***28.7*** ***(n = 5)***		***85.3*** ***(n = 30)***	***14.8*** ***(n = 5)***		***36.0*** ***(n = 30)***	***15.7*** ***(n = 5)***		***30.0*** ***(n = 30)***
	SD	71.3		86.6	17.8		68.8	6.5		35.1	3.33		8.42
	CV(%)	108.0		55.0	62.1		80.7	44.0		97.4	21.3		28.1

*Number of investigated samples.

Should these Ca concentrations reflect any trend in site-specific behavior of *Aneura pinguis* cryptic species A. three lineages? Preliminary observations may be indicative of the calciphilous character of A1, specifically for the PNN site, followed by A2 in the case of BS. Lineages identified at the relatively lower share of active Ca, that is below 1.00% may fall into the acidophilous range. The percentage share of active Mg into its total concentrations followed similar distribution patterns like active Ca, with A1 recording 2.53% at the PNN site. Magnesium and calcium are divalent elements, which significantly control the alkalinity of soil environment.

In the case of the current study, the occurrence of this lineage (i.e. A1) at this site is not a random process. By applying the same criteria like for active Ca, it appeared that A1, A2 and A3 at the Beskidy as well as Tatry Mts. met the rule of active Mg < 1.00%.

Potassium (K) and sodium (Na) have shared the most in their total content which was several times lower as compared mostly to total Ca. Despite this fact, active K represented from 10.6 to 24.8% whereas for Na, the values were significantly higher, i.e., 13.9 to 43.4%, even. It should be pointed out that these two monovalent elements are mostly responsible for regulating diffusion processes, hence their geochemical solubility raises; but this is not to alter the effects controlled by both active Ca and Mg.

### Interactions for lineages *versus* alkaline elements *versus* pH

Multiple comparisons of mean ranks in the ANOVA using the post hoc Scheffe tests showed statistically significant (p < 0.05) differences between growth sites of lineages A1, A2, A3 with respect to active forms of Ca, Mg, K, Na and pH of soil materials. The greatest similarity was observed for the site identified with lineages *A. pinguis* A1 and A2, but differed significantly for two factors, i.e. pH and Ca.

*A. pinguis* lineage A3 grew at the most distinct site. It differed statistically and significantly from the lineage A1 in terms of the total content of Ca, Mg and Na as well as active forms of Mg, K and Na. In the case of A2, the same statistical pattern was observed for total content of Ca, Mg and active forms of Mg, Na, respectively. The similarities and differences which emerged from statistical evaluation corroborated strictly those observed from genetic data (Fig. 3). This appears as an unquestionable proof of chemical characteristics of sites in the internal speciation of A. *pinguis* cryptic species A.

### Genetic lineage adaptability index *versus* site alkalinity and acidity (pH)

Data reported earlier have shown some trends in the site preference of the particular *A. pingui*s lineages growing at Pieniny (PNN), Beskidy (BS) and Tatry (T) Mts. Next, the concentrations of the alkaline elements (Ca, Mg, K, Na) in terms of their total as well as active forms should be supported by a comparative index applicable for any site. We have suggested the active forms of Ca/(Mg + K + Na), (Table 4) which should reflect the dynamic character of site reaction (pH) resulting from an equilibrium among these elements. Then, the growth response of genetic lineages A1, A2 and A3 has been considered to be strongly or weakly integrated to high or low values of this index. Its mean values along with pH are listed below:

**Table 4:** Global site specific index of active forms of alkaline elements (Ca, Mg, K, Na) and pH (alkalinity, acidity) in the growth media of *A. pinguis* cryptic species A genetic lineage A1, A2, A3 at Pieniny, Beskidy and Tatry Mts.

Site	Descriptive statistics	Genetic lineage A1		Genetic lineage A2		Genetic lineage A3	
		Ca/(Mg + K + Na)	pH	Ca/(Mg + K + Na)	pH	Ca/(Mg + K + Na)	pH
Pieniny Mts. (PNN)	Min	0.36	5.80				
	Max	6.70	8.10				
	***Mean***	3.24 (n = 25)*	***7.50 (n = 25)***				
	SD	1.37	0.57				
	CV(%)	42.3	7.60				
Beskidy Mts. (BS)	Min	2.15	7.89	0.017	7.63		
	Max	4.70	8.14	15.8	8.10		
	***Mean***	***2.70 (n = 10)***	***8.05 (n = 10)***	***4.34 (n = 15)***	***7.85 (n = 15)***		
	SD	0.74	0.08	3.77	0.15		
	CV(%)	27.7	1.04	86.9	1.96		
Tatry Mts (T)	Min	0.066	5.15			0.025	5.16
	Max	1.75	7.46			3.78	7.90
	***Mean***	***0.87 (n = 5)***	***6.45 (n = 5)***			***1.31 (n = 30)***	***7.08 (n = 30)***
	SD	0.79	0.99			0.91	0.54
	CV(%)	90.7	15.3			69.7	7.60

Lineage A1 index: PNN (3.24) > BS (2.70) > T (0.87).

Lineage A2 index: BS = 4.34.

Lineage A3 index: T = 1.31.

The respective pH values changed quite accordingly to the indices as shown below:

Lineage A1 site pH: BS (8.05) > PNN (7.50) > T (6.45).

Lineage A2 site pH: BS = 7.85.

Lineage A3 site pH: T = 7.08.

These ranges imply that genetic lineages A1 and A2 are by essence both calciphilous biotypes and may occur on sites rich in Ca, mostly alkaline as confirmed by the PNN and BS sites. On the other hand, some biotypes of the lineage A1 may be easily adapting also to low Ca concentrations, indicative of acidophilous features, as in the case of A3. Both (A1 and A3) occur at the Tatry MTs.

**Figure 4:**
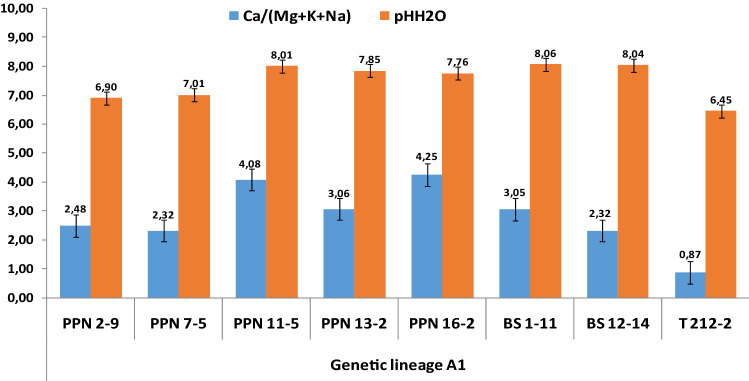
Singular site specific index of active forms of alkaline elements (Ca, Mg, K, Na) and pH in the growth media of *A. pinguis* cryptic species A genetic lineage A1 at Pieniny, Beskidy and Tatry Mts.

**Figure 5:**
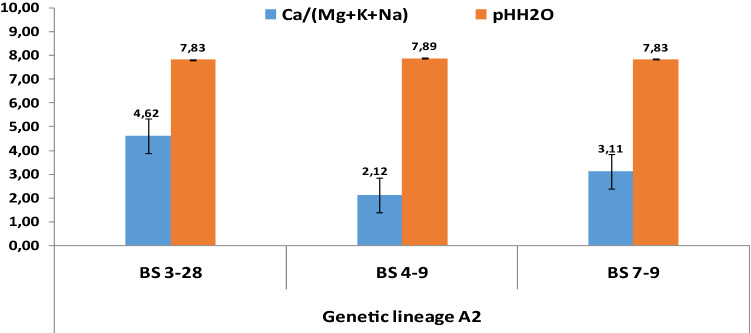
Singular site specific index of active forms of alkaline elements (Ca, Mg, K, Na) and pH in the growth media of *A. pinguis* cryptic species A genetic lineage A2 at Beskidy Mts.

**Figure 6:**
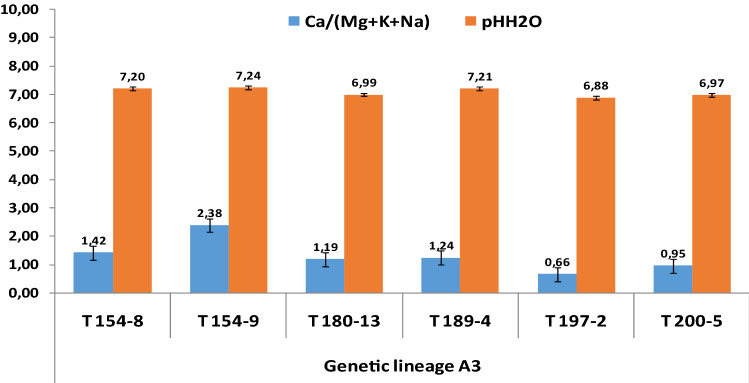
Singular site specific index of active forms of alkaline elements (Ca, Mg, K, Na) and pH in the growth media of *A. pinguis* cryptic species A genetic lineage A3 at Tatry Mts.

A detailed distribution of indices as well as respective pH is illustrated by the Figs. 4, 5 and 6, specifically for the genetic lineages A1, A2 and A3, respectively. The mean index values for the PNN site is 3.24 which discriminates the data into two groups: 60% < 3.24 and 40% > 3.24. In the case of BS, the mean value amounted to 2.70, but for only two sampling sites. Therefore, the mean values of the singular site specific index shows a clear pattern, which strengthens the preferential adaptation of A1 in prevalence to alkalinity as follows: PPN (3.24) > BS (2.70) > T (0.87).

The genetic lineage A2 outlines a great variability in terms of the site specific index, which was slightly high (4.62) only for the sampling site BS 3–28. It should be mentioned that the mean value at this site raised up to 4.34, hence being 56% lower than the highest and next 49% higher than the lowest index. Curiously, the respective pH values did not vary significantly (7.83–7.89), which implies that A2 is decidedly calciphilous.

Indices reported in the Fig. 6 fluctuated widely from 0.66 to 2.38 with a mean of 1.31. Only two values were higher but the remaining, i.e. about 67% placed below. Such high share reveals that the genetic lineage A3 is basically acidophilus. This is decidedly outlined by significantly low values of indices as a consequence of low concentrations of active Ca.

## Discussion

Growth characteristics and spatial distribution of bryophytes are evoked as mostly related to weather conditions. The case of genetic lineages (A1, A2, A3) within cryptic species of *A. pinguis* cryptic species A implies that some additional factors should be taken into consideration, particularly the levels of alkaline elements i.e., Ca, Mg, K, Na and the respective pH of the growth medium (soil, rock, peat).

Liverwort flora, *A. pinguis* species A as well as its cryptic forms among others, play an important ecosystem role due to their great capacity to acting effectively and successfully as soil binder and nutrient trapper. The latter feature seems to be strictly connected to the biological characteristics of soil crust expressed by Seitz et al.^23^ as biocrust. It should be mentioned, that the emergence of this biologically altered ecosystem is preconditioned by the natural composition of the growth substrate, particularly for the alkaline earth elements (Ca, Mg, K, Na). These are chemically in constant equilibrium with hydrogen (H) with pH as operational site indicator.

We developed a hypothesis strictly inherent with habitat preferential criteria for genetic lineages (A1, A2 and A3) of A*. pinguis* cryptic species A. They differ clearly (Fig. 3), however not enough to include them in separate species. In this study, the divergence between A1, A2 and A3 ranged from 0.20 to 1.0% in combined cpDNA sequences and in ITS. The interspecies divergence of 2% is often proposed in different taxa as a threshold between species^24^. However in some cases, arbitrary distance thresholds can suffer from varying rates of false-positive and false-negative error, depending on the data^25^.

It seems, that this genetic diversity is caused not only by geographical distance but also by other factors such as the mineral composition of the growth medium and its pH. Therefore all genetic lineages may share the same geographic region. This is visible especially in the Tatry Mts., where we can meet all 3 lineages.

**Figure 7:**
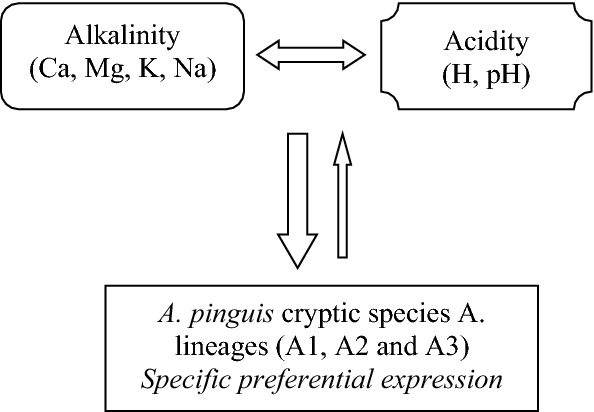
*Aneura pinguis* conceptual site-specific preferential expression towards alkalinity and acidity (Authors).

Data listed in the subsection *Genetic lineage adaptability index versus site alkalinity and acidity (pH)* as well as in Table 4 (with development by Figs. 4, 5 and 6) support the *A. pinguis* conceptual site-specific preferential (Fig. 7). The course of this process was described by Silva et al.^15^ as the species-area relationship (SAR), when concluding that not only microclimate can influence bryophyte richness, but opportunistic colonisation by bryophyte is also possible. This opportunistic colonisation has been reorientated in our study to the chemical composition of the ecological growth sites of *A. pinguis* lineages. The concentrations of water soluble (active) alkaline elements (Ca, Mg, K, Na), of which calcium particularly as well as the suggested site specific index i.e. Ca/(Mg + K + Na) enabled discriminating *A. pinguis* lineages: A1 and A2 exhibited decidedly both calciphilous biotypes and may occur on site rich in Ca, that is mostly alkaline as confirmed by the Pieniny (PNN) and Beskidy (BS) sites. On the other hand, some biotypes of the lineage A1 may be easily adapting also to low Ca concentrations, indicative of acidophilous features, as in the case of A3. It should be mentioned that both A1 and A3 occur at the Tatry MTs (T). The correlation calculated for the pairs Ca/(Mg + K + Na) and pH for the whole *A. pinguis* lineages showed r = 0.496 and was statistically significant at the level of p ≤ 0.01. This value explains at about 50% the potential role of this index in evaluating the complexity of the interactions involving A1, A2 and A3 lineages.

Field investigations on cryptic diversity in bryophyte soil-banks along a desert elevational gradient^16^ revealed even, that community compositions shifted with increasing elevation, suggesting that soil-banks are an important ‘cryptic’ component of the regional species pool. This explanation finds its strict argumentation in the fact that soil-banks are naturally rich in mineral alkaline elements from runoff deposition and hence “minerally" attract bryophytes, *A. pinguis* species among others, by creating favorable growth and colonisation conditions. Investigations carried out at the Reberce Nature Reserve (Western Carpathians, Poland)^26^, stated among others that 63% of terrestrial epixylic bryophytes, which represented the largest group occurred abundantly in the undergrowth, growing both on rich mineral soil and humus.

Earlier studies by Bates and Farmer^11^ have outlined some incentives for any research dealing with qualifying or quantifying potential effects of mineral elements in soil crust colonisation by bryophytes. The growth of *Calliergon cuspidatum* and *Pseudoscleropodium purum* from chalk soil was reduced when high Ca concentrations were top applied, whereas *Pleurozium schreberi* and *Pseudoscleropodium purum* from acidic clay remained unaffected. We learned from this response, that site adaptability of these lower plants is a discrete natural process driven mostly by chemical composition of growth substrate, particularly of alkaline minerals. Low or high concentrations of Ca, for instance in bryophytes and moss shoots, should not be strictly indicative of their calciphilous or acidophilous features. The same applies broadly to *A. pinguis* species and its relevant genetic lineages A1, A2 and A3, decidedly.

**Figure 8:**
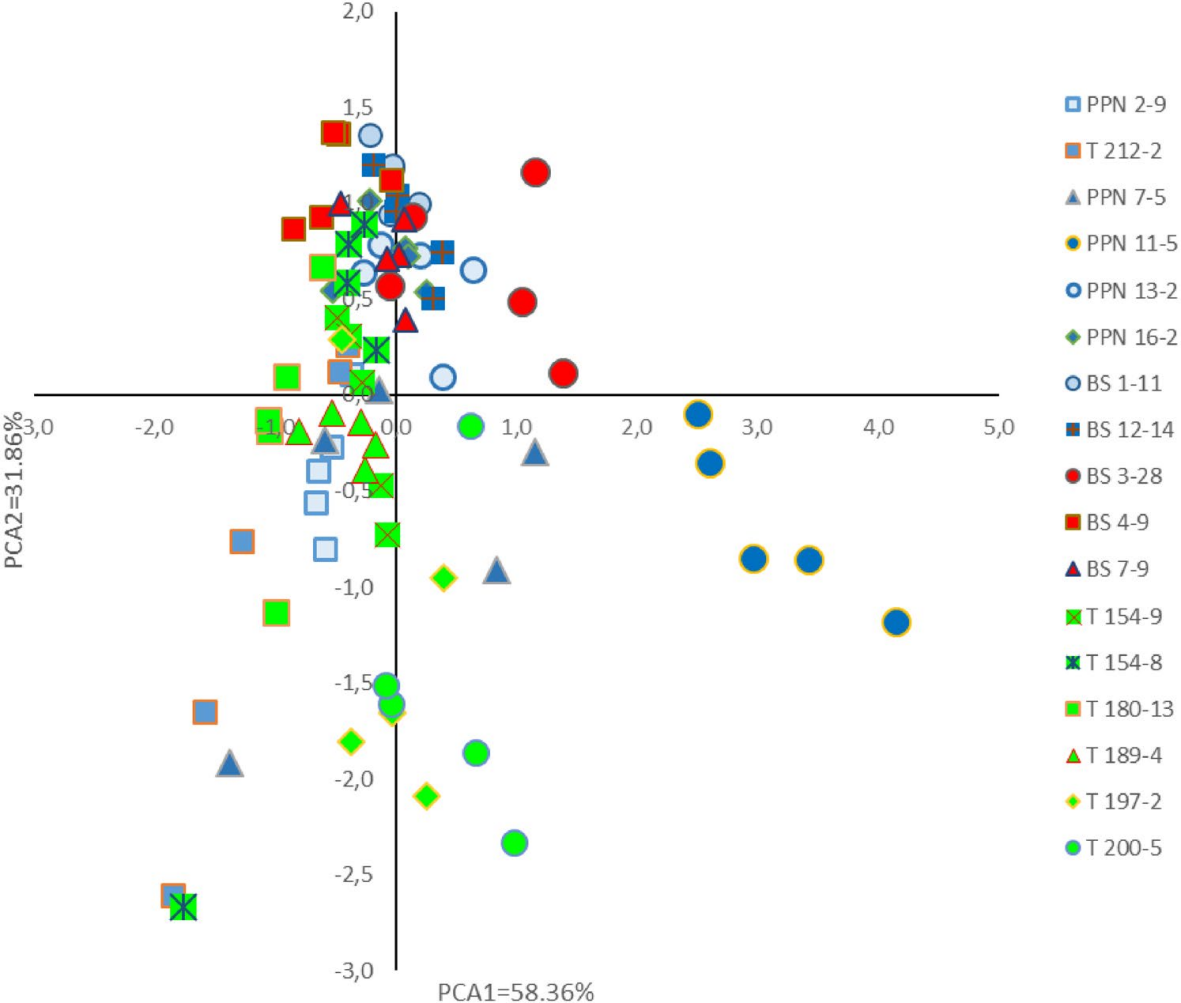
Distribution of samples in two principal components (PCA1 and PCA2) based on three chemical soil parameters: active Ca and Mg forms, pH).

Confirmations of these patterns were checked out by the principal component analysis (PCA) process (Fig. 8), where lineage assemblies and their focus or not to chemical parameters (Ca, Mg, K, Na) as well as Ca/(Mg, K, Na) with pH deserved special attention. The distribution of investigation sites, namely Pieniny Mts (PNN), Beskidy Mts (BS) and Tatry MTs (T) implies, that the lineages (A1, A2, A3) form a fairly compact group with positive PCA2 values. Taking into consideration PCA1 values, it can be seen that lineages assemble in the middle of the graph, while the PPN11-5 sample belonging to the blue sample group is separated from the others.

The correlations established between Ca, Mg, Ca/(Mg + K + Na) and pH with PCA1 (Table 5) are of high importance. The site concentrations of Ca, in some cases Mg too, are crucial in controlling pH, but this process should be shaped by each element individually. In another words, calciphilous or acidophilous *A. pinguis* species may be “remotely” attracted by high Ca (or Mg) site concentrations, i.e. alkaline pH or low Ca (or Mg), favorable for acidity emergence.

**Table 5:** Set of correlations coefficients between soil chemical parameters Ca, Mg and pH and two first principal components PCA1 and PCA2.

Soil chemical parameters	PCA1	PCA2
Ca	0.943	0.002
Mg	0.719	0.612
Ca/(Mg, K, Na)	− 0.870	0.126
pH	0.532	− 0.337

The site specific index Ca/(Mg, K, Na) exhibiting high but negative correlation with PCA1 is a proof that the colonisation of the investigated sites by *A. pinguis* species could have been performed on a specifically clear basis: either Ca or Mg, but not both equally. This finding shows that mineral elements responsible for alkalinity or acidity should be treated separately.

## Conclusions and statements

The preferential site-specific concept was verified on ecological zones hosting three lineages A1, A2, A3 of *A. pinguis* cryptic species A. The research revealed far-gone adaptability feature of these lineages, but related to site alkalinity and acidity (pH). We focused the concept on geochemical processes resulting from Ca, Mg, K and Na interactions with the ambient soil environment. Data have shown some trends in the site preference of the particular *A. pingui*s lineages growing at Pieniny (PNN), Beskidy (BS) and Tatry (T) Mts. The lineage adaptability index, i.e. Ca/(Mg + K + Na), which reflects the dynamic character of site reaction (pH) implied, that genetic lineages A1 and A2 are by essence both calciphilous biotypes and may occur on sites rich in Ca, that is mostly alkaline as confirmed by the PNN and BS sites. Some biotypes of the lineage A1 may be easily adapting also to low Ca concentrations, indicative of acidophilous features, as in the case of A3, both occurring at the Tatry MTs.

The site concentrations of Ca, in some cases Mg too, are crucial in controlling pH, but this process should be shaped by each element individually. In another words, calciphilous or acidophilous *A. pinguis* species may be “remotely” attracted by high Ca (or Mg) site concentrations, i.e. alkaline pH or low Ca (or Mg), favorable for acidity emergence. This finding shows, that mineral elements responsible for alkalinity or acidity should be treated separately. We also concluded, that mineral composition of soils and pH might initiated process of differentiation within *A. pinguis* cryptic species A, resulting in three genetic lineages A1, A2 and A3.
